# Transcriptional profiling reveals intrinsic mRNA alterations in multipotent mesenchymal stromal cells isolated from bone marrow of newly-diagnosed type 1 diabetes patients

**DOI:** 10.1186/s13287-016-0351-y

**Published:** 2016-07-12

**Authors:** Kalil A. de Lima, Gislane L. V. de Oliveira, Juliana N. U. Yaochite, Daniel G. Pinheiro, Júlia T. C. de Azevedo, Wilson Araujo Silva Jr, Dimas T. Covas, Carlos E. B. Couri, Belinda P. Simões, Julio C. Voltarelli, Maria C. Oliveira, Kelen C. R. Malmegrim

**Affiliations:** Center for Cell-Based Research, Regional Blood Center of Ribeirao Preto, Ribeirao Preto Medical, University of Sao Paulo, Ribeirao Preto, Brazil; Department of Biochemistry and Immunology, Ribeirao Preto Medical School, University of Sao Paulo, Ribeirao Preto, Brazil; Department of Clinical and Toxicological Analysis, Federal University of Ceará, Fortaleza, Ceara Brazil; Department of Clinical Medicine, Ribeirao Preto Medical School, University of Sao Paulo, Ribeirao Preto, Brazil; Department of Clinical, Toxicological and Bromatological Analysis, Faculty of Pharmaceutical Sciences of Ribeirao Preto, University of Sao Paulo, Ribeirao Preto, Brazil; Tenente Catao Roxo, 2501, Monte Alegre, 14051-140 Ribeirao Preto, Sao Paulo Brazil

**Keywords:** Mesenchymal stromal cells, Bone marrow, Type 1 diabetes, Transcriptome, Microarrays, Cell therapy

## Abstract

**Background:**

Bone marrow multipotent mesenchymal stromal cells (MSCs) are a diverse subset of precursors that contribute to the homeostasis of the hematopoietic niche. MSCs can be isolated and expanded in vitro and have unique immunomodulatory and regenerative properties that make them attractive for the treatment of autoimmune diseases, including type 1 diabetes (T1D). Whether autologous or allogeneic MSCs are more suitable for therapeutic purposes has not yet been established. While autologous MSCs may present abnormal function, allogeneic cells may be recognized and rejected by the host immune system. Thus, studies that investigate biological characteristics of MSCs isolated from T1D patients are essential to guide future clinical applications.

**Methods:**

Bone marrow-derived MSCs from recently diagnosed type 1 diabetes patients (T1D-MSCs) were compared with those from healthy individuals (C-MSCs) for morphological and immunophenotypic characteristics and for differentiation potential. Bioinformatics approaches allowed us to match absolute and differential gene expression of several adhesion molecules, immune mediators, growth factors, and their receptors involved with hematopoietic support and immunomodulatory properties of MSCs. Finally, the differentially expressed genes were collated for functional pathway enrichment analysis.

**Results:**

T1D-MSCs and C-MSCs were similar for morphology, immunophenotype, and differentiation potential. Our absolute gene expression results supported previous literature reports, while also detecting new potential molecules related to bone marrow-derived MSC functions. T1D-MSCs showed intrinsic abnormalities in mRNA expression, including the immunomodulatory molecules VCAM-1, CXCL12, HGF, and CCL2. Pathway analyses revealed activation of sympathetic nervous system and JAK STAT signaling in T1D-MSCs.

**Conclusions:**

Collectively, our results indicate that MSCs isolated from T1D patients present intrinsic transcriptional alterations that may affect their therapeutic potential. However, the implications of these abnormalities in T1D development as well as in the therapeutic efficacy of autologous MSCs require further investigation.

**Electronic supplementary material:**

The online version of this article (doi:10.1186/s13287-016-0351-y) contains supplementary material, which is available to authorized users.

## Background

Multipotent mesenchymal stromal cells (MSCs) are a diverse subset of precursors found in the stromal fraction of the bone marrow and other adult tissues, presenting osteogenic, adipogenic, and chondrogenic differentiating potentials [1–3]. Stromal cells are usually detected in perivascular areas and present immunomodulatory properties [1, 4]. They have therefore been explored as tools to modulate inflammatory response, induce peripheral tolerance, and promote tissue repair [5]. In addition, murine bone marrow MSCs are physically close to most hematopoietic stem cells (HSCs) and express high levels of genes related to HSC maintenance and retention, which makes them important contributors for maturation of the hematopoietic compartment [6–9].

MSCs can be expanded in vitro and have unique immunomodulatory and regenerative properties, which render them attractive for treatment of autoimmune and inflammatory disorders [10]. Initial studies revealed that bone marrow MSCs inhibited T-cell proliferation in vitro and were immunosuppressive in a model of skin allograft rejection [11–13]. In addition, bone marrow MSCs were shown to inhibit dendritic cell differentiation and B-cell proliferation, impair the cytolytic potential of natural killer cells, and increase T regulatory cell (Treg) differentiation and function [10, 14]. The therapeutic effects of MSCs are partially due to their ability to produce and secrete a vast array of soluble mediators and other molecules with immunomodulatory properties, such as hepatocyte growth factor (HGF), indoleamine 2,3-dioxygenase (IDO), cyclooxygenase-2 (COX2), IL-10, prostaglandin E2, nitric oxide, and transforming growth factor beta 1 (TGFβ-1) [14].

In the last decade, many studies have demonstrated the therapeutic potential of MSC transplantation in experimental models of autoimmune diseases, including type 1 diabetes (T1D) [15–18]. In 2006, Lee et al. [19] showed that human MSCs were able to migrate and promote pancreatic repair in nonobese diabetic/severe combined immunodeficiency (NOD/SCID) mice. Later, several other studies in experimental models of T1D have demonstrated that MSC transplantation delays the onset of disease or even reverses hyperglycemia [18–21]. Further investigations have shown modulation of the inflammatory response and expansion of Tregs, in consequence decreasing pancreatic infiltrates and improving endogenous insulin production [20–23]. Transdifferentiation is not considered a major therapeutic mechanism of MSCs in T1D. Instead, cytokine and soluble factor release may account for immunosuppressive, anti-inflammatory, and regenerative properties that abrogate the autoimmune response and stimulate the survival and proliferation of resident/progenitor pancreatic cells through paracrine pathways [24].

In-vitro expanded MSCs are able to escape the immune system when administered intravenously, and may be useful tools in the allogeneic transplantation setting [25]. Under resting conditions, MSCs express low levels of major histocompatibility complex (MHC) class I, and do not express MHC class II or co-stimulatory molecules, such as CD80, CD86, CD40, or CD40L [26, 27]. The modulatory activity of MSCs depends on a process of “licensing” promoted by proinflammatory cytokines, especially tumor necrosis factor (TNF) and interferon gamma (IFN-γ) [28–34]. In a murine model, MSCs treated with high IFN-γ levels before infusion became immediately activated and were able to suppress graft versus host disease (GvHD) more efficiently than a fivefold-greater number of MSCs that had not been pretreated [35]. Conversely, under low IFN-γ levels MSCs upregulate the expression of MHC class II, behave as antigen-presenting cells, and, in consequence, may be recognized by alloreactive cells after transplantation [36–40]. Accordingly, whether allogeneic MSCs persist in the tissues of immunocompetent hosts after transplantation still remains under debate [41].

In the field of human autoimmune diseases, results are frequently divergent and lack consistency. In patients with Crohn’s disease (CD) [42], rheumatoid arthritis (RA) [43], systemic sclerosis (SSc) [44], and multiple sclerosis (MS) [45], MSCs were shown similar to those from healthy controls. In other studies, however, abnormalities were described in MSCs isolated from patients with SSc [46, 47], systemic lupus erythematosus (SLE) [48, 49], MS [50], and psoriasis [51–54]. While the clinical relevance of such findings has not yet been established, currently there is weak support for either autologous or allogeneic MSC-based therapies.

In-vivo studies are also somewhat contradictory. In T1D patients, a small clinical trial showed modest but significant preservation of C-peptide levels after transplantation with autologous MSCs [55]. Accordingly, in animal models, murine MSCs from both healthy and diabetic animals were therapeutically effective [56, 57]. On the other hand, when murine bone marrow-derived MSCs were used to treat prediabetic NOD mice, onset of disease was delayed by MSCs isolated from BALB/c, but not by autologous MSCs [55]. These data indicate that available evidence is still not strong enough to support a recommendation and that more studies should be performed in order to fully establish advantages and weaknesses of autologous or allogeneic MSCs.

Further studies to investigate the genetic and biological profiles of MSCs isolated from patients with autoimmune diseases are still warranted. To the best of our knowledge, it remains unknown whether MSCs isolated from newly diagnosed T1D patients have a similar molecular profile compared with their healthy counterparts. For this purpose, we characterized the global gene expression of bone marrow MSCs isolated from healthy individuals (controls, C-MSCs) and newly diagnosed T1D patients (T1D-MSCs). Herein, we describe the absolute and differential gene expression of several molecules involved with immunomodulation and hematopoietic support in C-MSCs and T1D-MSCs.

## Methods

### Patients and controls

Twenty-one T1D patients of median age 16 (range 13–31) years, 71 % (*n* = 15) male, were enrolled in this study, having been diagnosed with T1D within the previous 6 weeks. All patients had positive serum levels of anti-glutamic acid decarboxylase (anti-GAD) antibodies, and did not report any previous episodes of diabetic ketoacidosis (Additional file 1: Table S1). The control group included 10 healthy subjects, 50 % male (*n* = 5), with median age 34 (range 19–48) years, who had already been enrolled as bone marrow donors for allogeneic transplantation, and who voluntarily agreed to donate an additional 5 ml of bone marrow aspirate for research. All procedures were approved by the Institutional Review Board (Research Ethical Committee of Clinical Hospital of Ribeirao Preto Medical School, CEP-HCRP-USP #10095/02) and written informed consent was obtained from all individuals before bone marrow aspiration. Bone marrow samples were obtained from T1D patients and healthy donors through needle aspiration of the iliac crest.

### Isolation and culture of bone marrow MSCs

Bone marrow aspirates were collected in the presence of EDTA, and mononuclear cells were separated by Ficoll-Hypaque (Amersham-Pharmacia, Uppsala, Sweden) gradient density separation. Subsequently, the mononuclear cell layer was harvested and washed twice in PBS. Cells were centrifuged and resuspended in alpha-Minimum Essential Medium (α-MEM; Gibco, Life Technologies, Grand Island, NY, USA) medium supplemented with 15 % fetal bovine serum (FBS; Thermo Scientific, Rockford, IL, USA), 100 μg/ml penicillin, 100 μg/ml streptomycin, and 2 mM l-glutamine (all from Gibco, Life Technologies). The cells were then seeded in 75 cm^2^ flasks and incubated at 37 °C in a humidified atmosphere containing 5 % CO_2_ (passage 0). After 24 h, nonadherent cells were removed by replacing the medium, and fresh medium was added. The culture was examined daily by phase-contrast microscopy and every 3 days the medium was partially changed to remove nonadherent cells and cell debris. When the cells reached at least 70–80 % confluence, they were detached using trypsin–EDTA 0.05 % (Gibco, Life Technologies) and passaged at a split ratio of 1:2 until the third or fourth passage.

### MSC morphology and immunophenotypic profiling

After expansion in culture until the third or fourth passage, C-MSCs and T1D-MSCs were assessed for morphology by inverted (Axiovert 40 CFL; Carl Zeiss, Goetingen, Germany) and light (TS100; Nikon, China) microscopy. C-MSCs and T1D-MSCs were then incubated with monoclonal antibodies against CD45, CD14, CD44, CD29, CD51/61, CD13, CD54, HLA-CLASS I (HLA-A/B/C), HLA-CLASS II (HLA-DRB1), CD90, KDR, CD34, CD49e, CD105, CD73, or STRO-1 (Becton-Dickinson (BD), San Jose, CA, USA) for 30 min in the dark. Immunophenotypic analysis was performed using FACSCalibur (BD) equipment and 20,000 cells were acquired and analyzed by FlowJo 10 software.

### Differentiation into mesoderm lineages

In-vitro adipogenic differentiation of C-MSCs and T1D-MSCs was induced using α-MEM medium supplemented with 15 % FBS, 100 mM dexamethasone (Prodome, Campinas, SP, Brazil), 10 μg/ml insulin (Sigma-Aldrich, Saint Louis, MO, USA), and 100 μM indomethacin (Sigma-Aldrich). MSCs cultured with α-MEM medium supplemented with 15 % FBS served as the negative control. Culture medium was changed every 3 days and cells were maintained in culture for 21 days. MSCs were then fixed with ethanol (70 %) and stained with Sudan II-Scarlate and Harris hematoxylin. The presence of lipid vacuoles in the MSCs was observed through light microscopy.

Osteoblastic differentiation was initiated by seeding MSCs in the presence of osteogenic differentiation medium composed of MSC growth medium; that is, α-MEM supplemented with 7.5 % FBS plus 1 M glycerol-2-phosphate (Sigma-Aldrich), 20 mM l-ascorbic acid (Sigma-Aldrich), and 0.1 mM dexamethasone (Sigma-Aldrich). The culture medium was replaced every 3 days during a period of 21 days. During the same period, control cells were kept in standard α-MEM with 7.5 % FBS (HyClone; Thermo Fisher Scientific Inc., USA).

All cells were fixed and stained by the von Kossa method, which indicates calcium deposition, subsequently analyzed with an Axioscope 2.0 Zeiss microscope equipped with an AxioCam HR camera (Carl Zeiss).

For chondrogenic induction, 10^6^ cell pellets were cultured for 3 weeks under chondrogenic medium containing 10 ng/ml of TGF-β3 (PeproTech, USA), 100 μM sodium pyruvate (Gibco, Carlsbad, CA, USA), 0.1 μM dexamethasone (Decadron, Brazil), 50 μM ascorbic acid, 0.5× ITS A (insulin–transferrin–selenium-sodium pyruvate A; Gibco), and 0.2 % human albumin (Aventis Behring, Australia) PeproTech in DMEM (Gibco). Cell pellets were harvested at 3 weeks post induction, fixed overnight with 4 % PFA, and sections were prepared for immunohistochemistry. For staining, sections were incubated with anti-type II collagen rabbit antibody (Novocastra™, Newcastle, UK), and subsequently stained with hematoxylin and eosin and analyzed with an Axioscope 2.0 Zeiss microscope equipped with an AxioCam HR camera (Carl Zeiss).

### Microarray analysis

Total RNA was isolated from random T1D-MSCs (*n* = 11) and C-MSCs (*n* = 10) by the Trizol method (Invitrogen, USA) and purified by RNeasy commercial kit (QIAGEN, USA) according to the manufacturers’ recommendations. RNA integrity was evaluated by microfluidic electrophoresis using Agilent 6000 RNA Nano chips and an Agilent 2100 Bioanalyzer (Agilent Technologies, Santa Clara, CA, USA). Only RNA samples that were free of proteins and phenol and that featured an RNA Integrity Number (RIN) ≥ 9.0 were used. Random RNA samples of T1D-MSCs and C-MSCs were selected (*n* = 4 for each group) and the global gene expression was analyzed by the One-color Microarray-Based Gene Expression Analysis Protocol system (Agilent Technologies) on glass slides with four microarrays of 44,000 probes each (4 × 44 k). The preprocess and statistical microarray analyses were performed using algorithms available in the R platform (R Foundation, Vienna, Austria) through the Linear Models for Microarray Data (LIMMA) package [56]. Heatmaps were generated by the HeatMapViewer module of GenePattern 2.0 software [57]. Genes with *p* < 0.05 and fold change (FC) > 2.0 were considered differentially expressed. Microarray data were deposited in the public database ArrayExpress (http://www.ebi.ac.uk/arrayexpress [ArrayExpress:E-MTAB-2976]).

### Absolute and differential expression of predefined gene categories

Encoding genes for collagens, integrins, and laminins, as well as for cytokines, growth factors, and chemokines, and their respective receptors, were classified according to the binary relationships of biological entities (BRITE) category available in the Kyoto Encyclopedia of Genes and Genomes (KEGG) database [58]. Different categories were represented by heatmaps generated by the HeatMapViewer module of the GenePattern 2.0 software [57]. Genes with multiple probes were represented by the median value. For absolute gene expression representation, genes in each category were sorted in ascending order, according to average value expression of the control group. Highly expressed genes had absolute gene expression value (EV) > 120. Only genes with *p* < 0.05 and FC > 2.0 were included in the heatmaps for differential gene expression.

### Functional enrichment analysis

The functional enrichment analysis was performed using DAVID software (Database for Annotation, Visualization and Integrated Discovery; National Cancer Institute at Frederick, Frederick, MD, USA) [59, 60]. The differentially expressed official gene symbols (upregulated and downregulated) were imported to the program and the Functional Annotation Chart module was used. In this module, we performed a standard ontology (GO FAT) available for all categories of the Gene Ontology database (biological process, molecular function, and cellular component), as well as analysis of the KEGG pathways database. Categories and pathways with *p* > 0.05 were considered statistically significant (Benjamini correction).

### Gene set enrichment analysis

Gene set enrichment analysis (GSEA) was performed using the GSEA 2.07 software (Broad Institute from Massachusetts Institute of Technology, MIT, Cambridge, MA, USA). The differentially expressed probes (*p* < 0.05) were imported to the program, and gene ontology and KEGG pathway analyses were performed with 1000 permutations (gene set type) for *p*-value calculation. Default parameters were used, except for “collapsing mode for probe sets = > 1 gene”, in which the median value was used. Categories with nominal *p* < 0.05 and false discovery rate (FDR) < 0.25 were considered statistically significant.

### Functional pathway analysis

To assess biological relationships among genes, the gene list with *p* < 0.05 and FC > 2.0 were ascertained using the Ingenuity Pathway Analysis (IPA) software (Ingenuity System, Redwood City, CA, USA; http://www.ingenuity.com), which assigns a *p* value to each network, according to the degree of overrepresentation of input genes as compared with the Ingenuity Pathways Knowledge database.

### Real-time PCR

cDNA was synthesized from different RNA samples used for microarrays (T1D-MSCs, *n* = 7 and C-MSCs, *n* = 6) using 200 ng of total RNA (High-Capacity cDNA Reverse Transcription Kit; Life Technologies, USA). For microarray validation, real-time PCR was performed with the Taqman Gene Expression Assay (Applied Biosystems, USA) according to the manufacturer’s protocol. Target gene expression was calculated using the comparative method for relative quantification after normalization to GAPDH gene expression: *GAPDH*, Hs02758991_g1; *CXCL12*, Hs03676656_mH; and *VCAM1*, Hs01003372_m1.

### In-vitro migration assay

In-vitro migration was assayed by transwell chamber (BD, Franklin Lakes, NJ, USA) with 8 μm porosity polyethylene terephthalate (PET) membrane. C-MSCs (*n* = 5) and T1D-MSCs (*n* = 5) were seeded in the upper layers of membranes with 100 μl of α-MEM medium without FBS. One chamber was inserted into each well of a 24-well plate filled with 600 μl of α-MEM supplemented with 50 % FBS. MSCs were incubated for 6 h at 37 °C. Cells that passed through the membrane pores to the underside were counterstained by Giemsa (Sigma-Aldrich). Nonmigrated cells were scraped off the upper surface of the membrane with a cotton swab. For each membrane, the number of migrated MSCs was counted in seven different fields using light microscopy.

## Results

### T1D-MSCs present typical morphology, immunophenotype, and mesodermic lineage differentiation

**Fig. 1:**
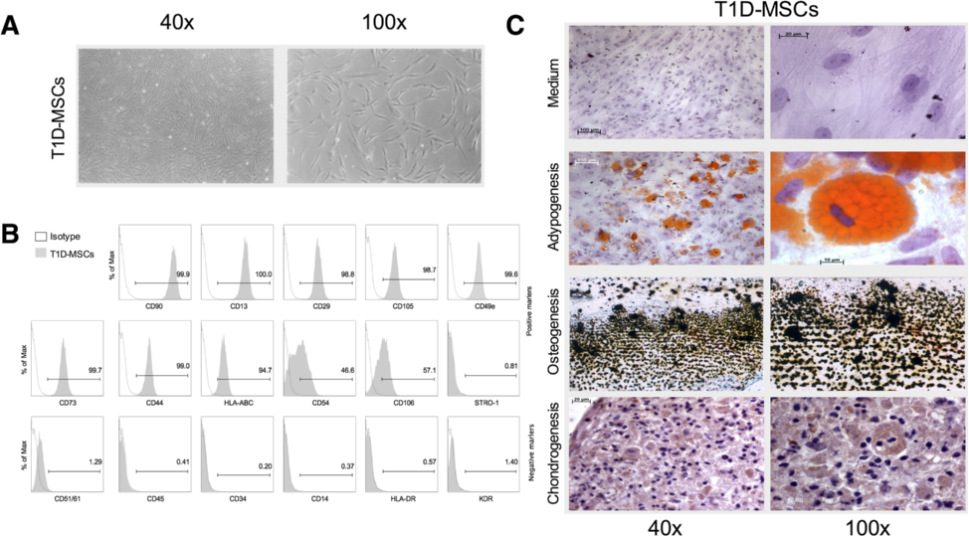
In-vitro expanded MSCs from type 1 diabetes patients (*T1D-MSCs*) show typical spindle-shaped morphology, mesodermic differentiation potential, and immunophenotypical profile. **a** Morphological characterization of T1D-MSCs at 40× (*left*) and 100× (*right*) magnification. **b** Representative histograms of positive and negative MSC surface markers. **c** Adipogenic, osteogenic, and chondrogenic differentiation of T1D-MSCs at 40× (*left*) and 100× (*right*) magnification. *MSC* multipotent mesenchymal stromal cell

T1D-MSCs or C-MSCs were morphologically and immunophenotypically characterized in vitro. We did not observe differences between C-MSCs and T1D-MSCs concerning proliferative capacity or doubling time (data not shown). In general, MSCs isolated from both groups achieved 70–80 % confluence every 7 days. MSCs appeared as a typical monolayer of spindle-shaped fibroblast-like cells and demonstrated ability to adhere to plastic during in-vitro expansion. At the third passage, T1D-MSCs were morphologically similar to C-MSCs (Fig. 1a). No significant difference between T1D-MSCs and C-MSCs was observed in the expression of typical MSC markers. Both MSC populations were positive for CD90, CD13, CD29, CD105, CD49e, CD73, CD44, HLA-ABC, CD166, CD54, CD106, and STRO-1, and were negative for the hematopoietic/endothelial markers CD51/61, CD45, CD34, CD14, HLA-DR, and KDR (Fig. 1b, Additional file 2: Table S2). Furthermore, cultured T1D-MSCs and C-MSCs, under specific stimuli, were able to differentiate towards adipogenic, osteogenic, and chondrogenic lineages (Fig. 1c).

### Transcriptional profile of T1D-MSCs is distinct from their healthy counterparts

To investigate whether T1D-MSCs present transcriptome abnormalities and to better understand molecular pathways that may regulate T1D-MSC biology, we performed a global gene expression analysis by microarray.

**Fig. 2:**
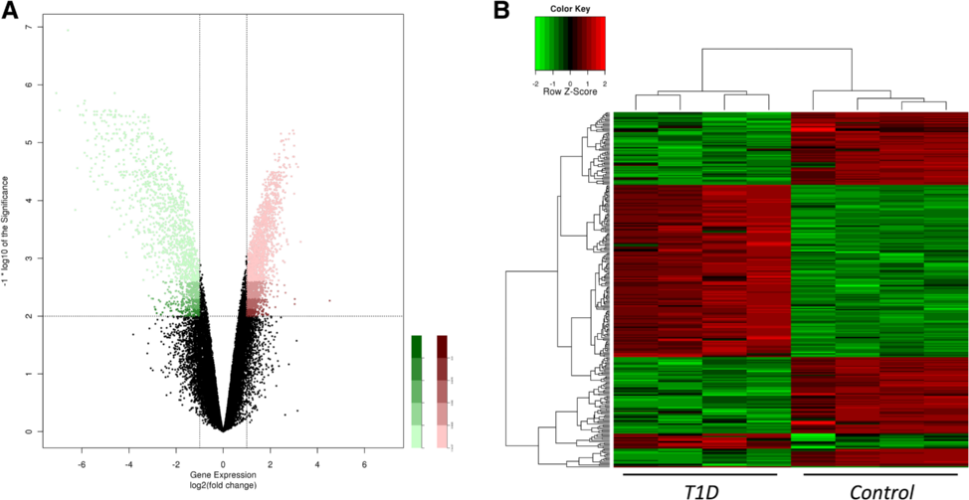
Distinct global gene expression in T1D-MSCs and C-MSCs. A total of 2149 genes were differentially expressed between T1D-MSCs (*n* = 4) and C-MSCs (*n* = 4). T1D-MSCs showed 1515 upregulated genes and 634 downregulated genes (FC > 2, *p* < 0.01, Student *t* test, Benjamini Hochberg correction). **a** Volcano plot of differentially expressed probes between T1D-MSCs and C-MSCs. Each plot represents one probe. Upregulated probes in T1D-MSCs are shown in *red* and downregulated probes in *green*. **b** Supervised clustering of differentially selected probes. *T1D* type 1 diabetes

Unsupervised clustering analysis showed distinctive gene expression signatures comparing T1D-MSCs to C-MSCs (Additional file 3: Figure S1) and we observed differential expression of 2978 probes between the groups (FC > 2, p < 0.05). Most of these probes were found upregulated in T1D-MSCs, when compared with C-MSCs (1926 upregulated and 1052 downregulated probes) (Fig. 2).

Aside from their role as structural elements, MSCs serve as resident sentinels that, upon activation, express surface molecules and produce soluble factors, coordinating tissue regeneration and inflammatory responses [4, 61]. In order to characterize the gene expression of several adhesion molecules, immune mediators, growth factors, and their receptors in bone marrow-derived MSCs from T1D patients and healthy controls, we first determined which mRNAs were more intensively expressed, using absolute gene expression analysis. Then, we specifically investigated which of these molecules were differentially expressed between T1D-MSCs and C-MSCs.

### VCAM-1 and other adhesion-related molecules are differentially regulated in T1D-MSCs

**Fig. 3:**
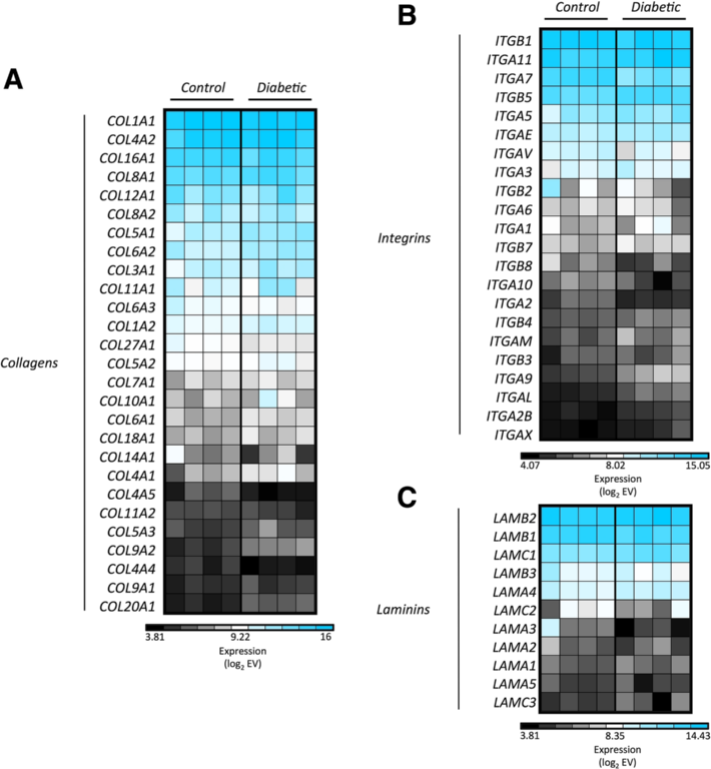
MSCs show high absolute gene expression of adhesion-related molecules. Absolute expression of genes encoding **a** collagens, **b** integrins, and **c** laminins in MSCs from healthy donors (*Controls*) and T1D patients (*Diabetic*). Genes with multiple probes were represented by the median value. The absolute gene EV was log_2_-transformed, and *blue* indicates high expression

Cultured MSCs from both groups (T1D-MSCs and C-MSCs) presented increased absolute mRNA expression of collagens, integrins, laminins, and other molecules related to extracellular matrix (ECM) maintenance, cell–cell adhesion, and cell–ECM interaction (EV > 120). Genes encoding type I, IV, V, VI, and VIII collagens were overexpressed (Fig. 3a), as well as those for CD29 (*ITGB1*), CD51 (*ITGAV*), and CD49a (*ITGA1*) (Fig. 3b). A similar increase was observed for codification of the three types of laminin chains, especially the β chain (Fig. 3c), and for other adhesion-related genes (absolute expression), such as *VCAM1*, *THY1*, and *CD44* (Additional file 4: Figure S2).

**Fig. 4:**
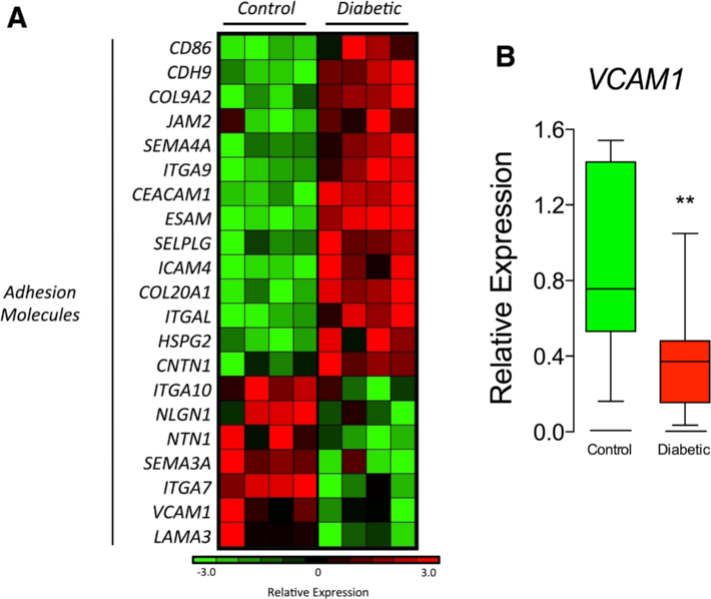
VCAM-1 and other adhesion-related molecules are differentially regulated in T1D-MSCs. **a** Heatmap of adhesion-related genes differentially expressed between MSCs from healthy donors (*Controls*, *n* = 4) and T1D patients (*Diabetic*, *n* = 4) (FC > 2, *p* < 0.05, Student *t* test, Benjamini Hochberg correction). Upregulated genes are shown in *red* and downregulated genes in *green*. **b** Relative expression of *VCAM1* by real-time PCR (Diabetic, *n* = 7; Control, *n* = 6). Mann-Whitney *U* test, *p* = 0.0041. *VCAM-1* vascular cell adhesion protein 1

Bioinformatics analysis was used to identify adhesion-related genes that were differentially expressed between T1D-MSCs and C-MSCs (FC > 2.0, *p* < 0.05). *VCAM1* was found downregulated in T1D-MSCs, and differences were also detected for expression of *SEMA4A*, *ITGA7*, *ITGB1*, and *LAMA3* (Fig. 4a). Microarray analysis was validated by quantitative real-time PCR, confirming that *VCAM1* was downregulated in T1D-MSCs (Fig. 4b).

### CXCL12, CCL2, and other chemotaxis-related molecules are differentially regulated in T1D-MSCs

**Fig. 5:**
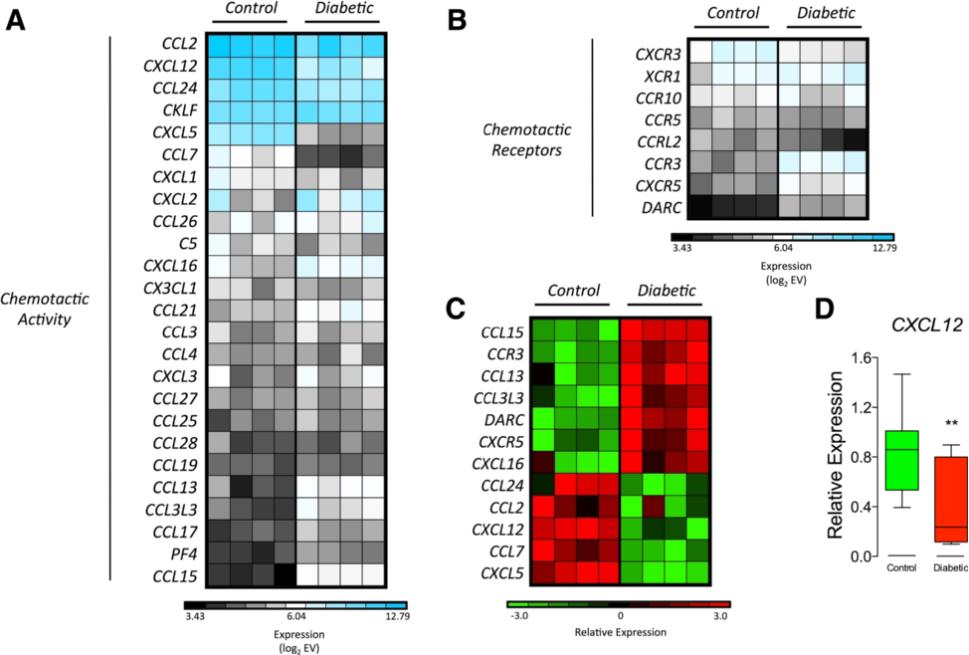
*CXCL12*, *CCL2*, and other migration-related molecules are differentially regulated in T1D-MSCs. Absolute gene expression of genes encoding **a** chemokines and **b** chemokine receptors. Genes with multiple probes were represented by the median value. The absolute gene expression value (*EV*) was log_2_-transformed, and *blue* indicates high expression. **c** Heatmap of the migration-related genes differentially expressed between MSCs from healthy donors (*Control*, n = 4) and T1D patients (*Diabetic*, n = 4) (FC > 2, *p* < 0.05, Student *t* test, Benjamini Hochberg correction). Upregulated genes are shown in *red* and downregulated genes in *green*. **d** Relative expression of *CXCL12* by real-time PCR (Diabetic, *n* = 7; Control, *n* = 10). Mann-Whitney *U* test, *p* = 0.0054

MSCs are able to migrate to sites of inflammation and to regulate the traffic of different hematopoietic cells. Chemokines and their receptors are key molecules for such activities [62]. Thus, we determined the absolute gene expression of chemokines and chemotaxis-related molecules in T1D-MSCs and C-MSCs. Our analyses detected increased absolute expression of genes encoding CXCL12, CCL24, chemokine-like factor (CKLF), CXCL5, and, especially, CCL2 (MCP-1) in MSCs from both groups (Fig. 5a). Among the chemokine receptors, *CXCR3* was mostly expressed (Fig. 5b). Additionally, *CXCL12*, *CCL2*, *CCL24*, and *CXCL5* were downregulated in T1D-MSCs compared with C-MSCs (Fig. 5c). Downregulation of *CXCL12* was confirmed by real-time PCR (Fig. 5d). Furthermore, despite having low absolute expression (EV < 120), genes encoding the chemokines CCL13, CCL15, CXCL16, and CCL3L3 and receptors CCR3, CXCR5, and Duffy antigen/chemokine receptor (DARC) were upregulated in T1D-MSCs compared with their healthy counterparts. Conversely, *CCL7* was downregulated (Fig. 5c).

### Differential expression of genes encoding cytokines, growth factors, cytokine/growth factor receptors, and matrix metallopeptidases

**Fig. 6:**
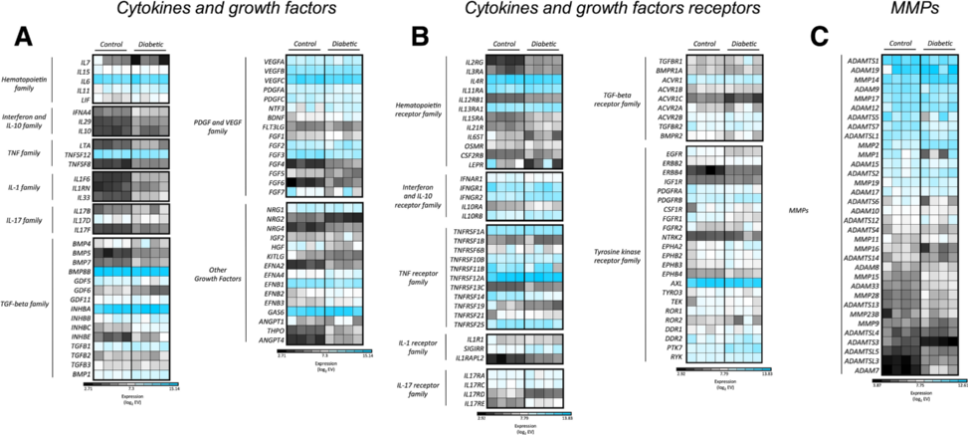
MSC absolute gene expression of cytokines, growth factors, receptors, and MMPs corroborates previous literature reports. Absolute gene expression of genes encoding **a** cytokines and growth factors, **b** cytokine/growth factor receptors, and **c** metalloprotease-related molecules. Genes with multiple probes were represented by the median value. The absolute gene expression value (*EV*) was log_2_-transformed, and *blue* indicates high expression. T1D-MSCs (*Diabetic*; *n* = 4) and C-MSCs (*Control*; *n* = 4). *MMP* matrix metallopeptidase. *IL* interleukin, *PDGF* platelet-derived growth factor, *TGFβ* transforming growth factor beta, *TNF* tumor necrosis factor, *VEGF* vascular endothelial growth factor

Cytokines and matrix metallopeptidase (MMP)-related molecules play a fundamental role in MSC-mediated immune regulation, in tissue regeneration, and also in MSC migration [62]. These functions are also associated with the expression of receptors on the surface of the MSCs, including the “licensing” receptors for IFN-γ and TNF-α [29]. In our analysis, both C-MSCs and T1D-MSCs presented increased absolute expression of genes encoding cytokines (IL-6, TGF-β1), growth factors (platelet-derived growth factor (PDGF), vascular endothelial growth factor (VEGF), fibroblastic growth factor (FGF), HGF) and MMP-related molecules such as MMP-2, MMP-9, and MT1-MMP (Fig. 6).

**Fig. 7:**
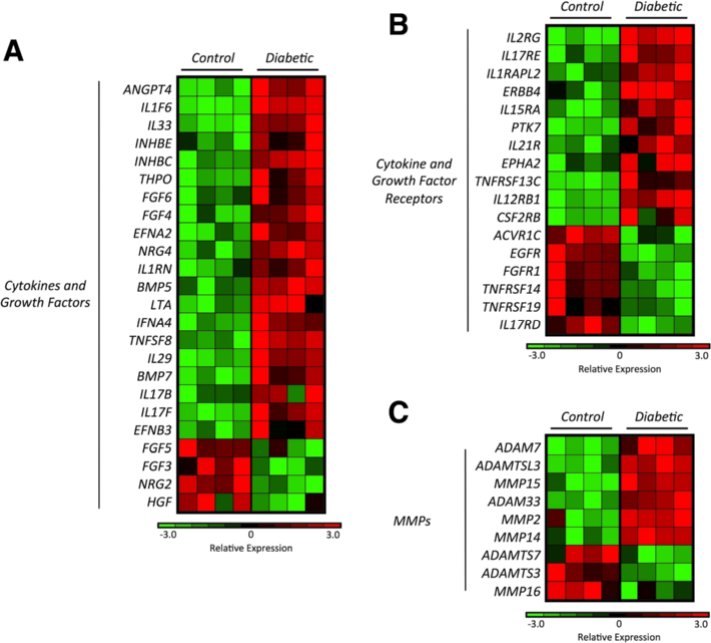
T1D-MSCs show distinct expression of genes encoding cytokines, growth factors, cytokine/growth factor receptors, and MMPs. Heatmap of **a** cytokines and growth factors, **b** cytokine/growth factor receptors, and **c** MMP genes differentially expressed between T1D-MSCs and C-MSCs (FC > 2, *p* < 0.05, Student *t* test, Benjamini Hochberg correction). Upregulated genes are shown in *red* and downregulated genes in *green*. T1D-MSCs (*Diabetic*; *n* = 4) and C-MSCs (*Control*; *n* = 4). *MMP* matrix metallopeptidase

Further analysis revealed 24 genes encoding cytokines or growth factors, 17 receptors for these molecules, and nine MMP-related proteins differentially expressed between T1D-MSCs and C-MSCs. Of these, *FGF3* and *HGF* (Fig. 7a), as well as *EGFR* and *FGFR1* (Fig. 7b), were downregulated in T1D-MSCs, while MT1-MMP (*MMP14*) and *MMP2* were upregulated in T1D-MSCs (Fig. 7c), when compared with their healthy counterparts. Both *MMP14* and *MMP2* are related to MSC migratory capacity. Indeed, we observed higher migratory capacity in MSCs from T1D patients than in those from healthy controls (Additional file 5: Figure S3).

### Hyperactivation of sympathetic nervous system signaling in T1D-MSCs

**Fig. 8:**
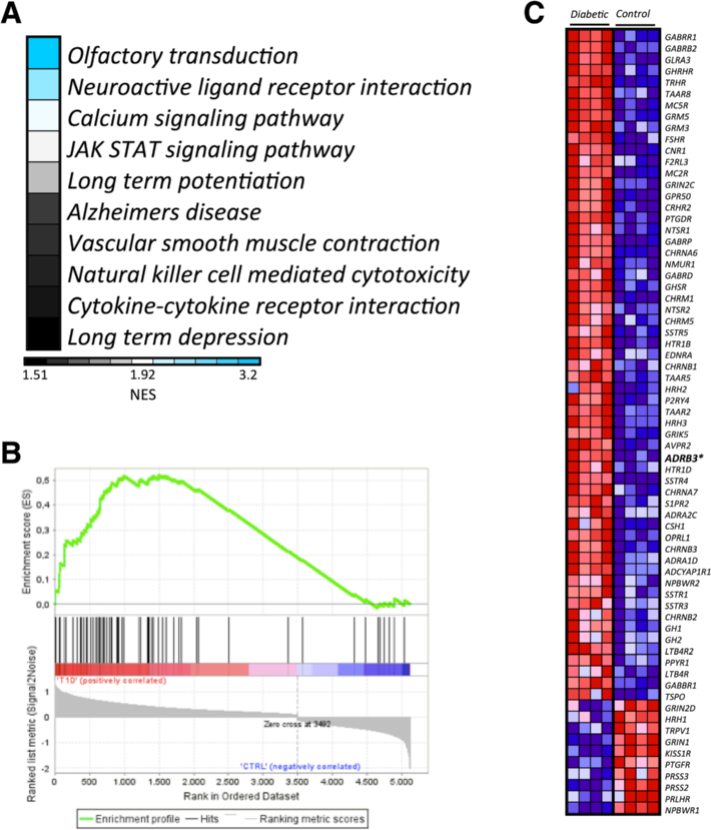
Pathway analysis suggesting hyperactivation of sympathetic nervous system signaling in T1D-MSCs. GSEA analysis of differentially expressed probes between T1D-MSCs and C-MSCs. **a** Enrichment analysis in KEGG pathways positively correlated with T1D-MSCs relative to C-MSCs; *blue* indicates KEGG pathways with higher normalized enrichment score (*NES*). Only data sets with *p* < 0.05 and FDR > 0.25 are shown. **b** Enrichment plot of the neuroactive ligand–receptor interaction KEGG pathway: *green curve* indicates the enrichment score (*ES*); *black vertical dashed lines* specify the maximum enrichment score. **c** Heatmap showing expression of genes in the leading edge subsets. The *ADRB3* gene is shown in *bold. Diabetic* T1D-MSCs, *Control* C-MSCs

Signature sequence probe lists were analyzed by collating genes into functional pathways in the KEGG database and ranking those pathways on the basis of statistical overrepresentation with the DAVID bioinformatics database and GSEA. DAVID analysis of the upregulated genes revealed enrichment of the neuroactive ligand–receptor interaction canonical pathway in T1D-MSCs (Additional file 6: Table S3). The same pathway was also significantly and positively correlated with T1D-MSCs in the GSEA analysis (Fig. 8a,b). The neuroactive ligand–receptor interaction pathway signaling is triggered by activation of G protein-coupled receptors that indirectly regulate opening and closing of ion channels after neurotransmitter binding [63]. When examining genes contained in this pathway, we found important upregulation of the β_3_-adrenergic receptor-encoding gene (*ADRB3*), suggesting activation of the adrenergic system in T1D-MSCs (Fig. 8c).

**Fig. 9:**
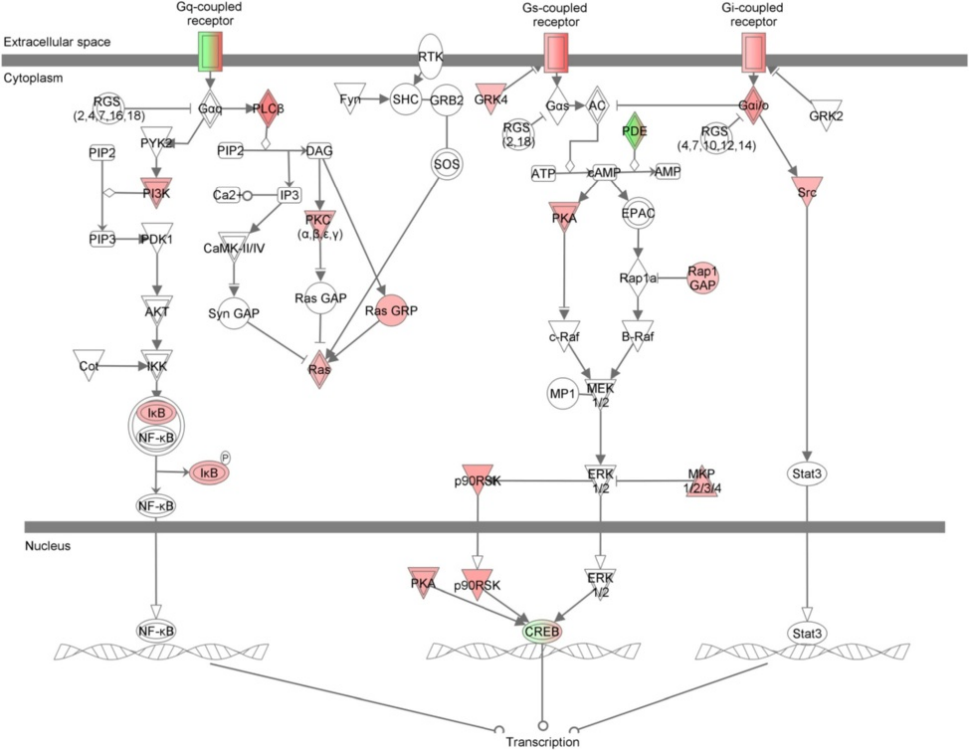
Pathway analysis using IPA software suggests β_3_-adrenergic receptor signaling activation in T1D-MSCs, with upregulation of several downstream molecules

To further investigate the hyperactivation of the adrenergic system, we then analyzed the expression of downstream genes involved in the β_3_-adrenergic signaling. For this purpose, we imported the data of differentially expressed probes into the IPA software and, as expected, most of the G protein-coupled receptor canonical pathway genes were upregulated (Fig. 9).

## Discussion

In the bone marrow, MSCs are in close contact with HSCs and express several factors responsible for the innate and adaptive immune cell maturation and support [64]. Additionally, in-vitro expanded MSCs isolated from bone marrow samples are able to modulate the immune system and have been investigated as treatment for several immune-mediated disorders [5]. However, it remains a matter of debate whether autologous or allogeneic MSCs are more suitable for therapeutic purposes in this context. On one hand, functionally compromised autologous MSCs may be therapeutically ineffective. On the other, allogeneic MSCs may be rejected by the host competent immune system [41].

Here, we demonstrate that in-vitro expanded T1D-MSCs present similar morphology, immunophenotype, and multipotential differentiation when compared with MSCs derived from healthy controls. However, our transcriptome analysis revealed more than 2000 probes differentially expressed between T1D-MSCs and C-MSCs.

Cellular function is a consequence of the interaction between DNA sequence, epigenetic status, mRNA and miRNA expression, and protein content, among other components. Although gene expression analysis offers limited information and some reports have shown notoriously poor correlation between mRNA and protein expression levels, it has been described that differentially expressed mRNAs correlate significantly better with their protein product than nondifferentially expressed mRNAs [65]. Thus, although in our study an absence of validation by other methods or functional experiments is a limitation, we believe that global transcriptomic analysis is a strong enough method to identify markers, including molecular pathways, surface receptors, and secreted factors, for further detailed studies.

Studies addressing potential abnormalities in MSCs derived from patients with autoimmune or inflammatory disorders are scarce and somewhat contradictory. While some authors report phenotypic, proliferative, and genetic abnormalities in cells derived from diseased patients, others describe them as nondifferent from their healthy counterparts [45–54]. Expressions of the TGF-β receptor gene and protein were found defective in MSCs from SSc patients [46], and *IL-6* and *IL-7* mRNA were downregulated in MSCs from SLE patients [48]. Mesenchymal cells derived from the skin of psoriasis patients produced more angiogenic and proinflammatory mediators and showed reduced antioxidant capacity when compared with those isolated from control specimens [51]. Microarray and genome-wide promoter methylation analyses revealed that skin-derived MSCs from psoriasis patients presented aberrant proliferative activity and increased apoptosis rates, markedly different from healthy control MSCs [52–54]. Recently, our group demonstrated genetic and functional alterations in MSCs isolated from MS patients (MS-MSCs) [50]. MS-MSCs in culture presented a senescent phenotype and reduced antiproliferative potential. In addition, microarray analysis showed 618 differentially expressed genes, including downregulation of *TGFB1* and *HGF*. Together, these results indicate that alterations may be disease specific and that abnormalities can involve different aspects of MSC function or morphology.

To provide better understanding of the potential molecules expressed by MSCs and to evaluate which of these were differentially expressed between T1D-MSCs and C-MSCs, we matched the absolute and differential gene expression of several adhesion molecules, immune mediators, and growth factors and their receptors. Such analyses allowed us to identify the most relevant differentially expressed molecules, considering the biology of MSCs. Our gene expression data corroborated previous reports, while revealing new molecules potentially related to bone marrow MSC function. As expected, MSCs from both groups of patients (T1D-MSCs and C-MSCs) presented high absolute expression of several well-described adhesion molecules, such as collagen VI [66], laminin-5 [67], integrins [68], fibronectin-1, and intercellular adhesion molecule-1 (ICAM-1) [69]. Additionally, in accordance with the literature, other important molecules were also found highly expressed in our analysis, such as VEGF [70–73], PDGF [73], FGF-2 [74, 75], HGF [70, 72, 76, 77], and the licensing receptors for IFN-γ and TNF [29, 31, 40, 78–80].

Focusing on the differential gene expression of immunoregulatory molecules between T1D-MSCs and C-MSCs, we found that vascular cell adhesion molecule-1 (VCAM-1) was downregulated in MSCs from T1D patients. Previous reports have shown that under inflammatory stimuli, MSCs produce large amounts of chemokines and upregulate VCAM-1 expression, which interacts with very late antigen-4 (VLA-4), rendering MSCs more adhesive to activated T cells [29]. This close proximity is pivotal for the immunosuppressive effect of MSCs [31]. Our results therefore suggest that this cell contact-dependent suppressive function of MSCs may be impaired in T1D patients.

We observed downmodulation of the *HGF* gene in T1D-MSCs. Indeed, HGF is also less expressed by bone marrow MSCs isolated from MS patients [50]. This pleiotropic cytokine that binds the tyrosine kinase transmembrane receptor c-Met [81] is associated with angiogenesis and cell survival [82, 83]. Injections of HGF stimulate kidney and liver regeneration, and prevent the onset of renal failure [84]. In experimental autoimmune encephalomyelitis (EAE) models, HGF secreted by MSCs promotes neural cell development and remyelination [85]. Interestingly, HGF is also suggested to be a cytoprotective factor for pancreatic β cells [21, 86, 87], and streptozotocin (STZ)-induced insulitis is observed to a higher degree in c-Met null mice than in wild-type littermates [88]. The HGF/c-Met signaling pathway is considered important for β-cell protection and proliferation in conditions of metabolic stress [89]. Thus, the observed downregulation of *HGF* in MSCs from T1D patients may indicate a decreased potential for pancreatic regeneration. Additionally, genes encoding the receptors epidermal growth factor receptor (EGFR) and fibroblastic growth factor receptor (FGFR) were also found downregulated in our T1D-MSCs. These receptors regulate stemness, and inhibit senescence, and are essential for cell growth, tissue repair, and homeostasis [90, 91]. Interestingly, EGFR signaling increases secretion of HGF [90, 92, 93]. We believe that downmodulation of *HGF* in T1D-MSCs may be a result of decreased EGFR signaling.

Moreover, membrane type 1-matrix metalloproteinase (MT1-MMP) and MMP2, whose encoding genes were found upregulated in T1D-MSCs, are essential for the invasive capacity of MSCs [94–102]. Interestingly, T1D-MSCs present higher migration capacity than C-MSCs. On the other hand, we detected downregulation of genes encoding CCL2, which is an important regulator of bone marrow monocyte emigration [103]. In EAE mice, the metalloproteinase-mediated paracrine proteolysis of CCL2 is key for the efficacy of murine MSC-based therapy. Indeed, MSCs that are CCL2-deficient have impaired ability to suppress IL-17 production by activated T cells and, in consequence, lose their protective effect [104, 105]. It has been described that MSCs cultured alone without cytokines produce minimal amounts of chemokines, and that unprimed MSCs cannot attract T cells [29]. Of note, our study analyzed MSCs after culturing and we were not able to determine whether these changes are related to culturing conditions beyond their exposure to the altered diabetic bone marrow milieu. How these gene expression alterations may affect therapeutic efficacy of bone marrow MSCs of T1D patients remains unknown and further functional experiments are still warranted.

Finally, to identify previously unknown immunologically relevant pathways associated with T1D-MSCs, we uploaded the differentially expressed genes into DAVID and GSEA bioinformatics databases. We detected preferential enrichment of canonical pathways related to sympathetic hyperactivity and also upregulation of the β_3_-adrenergic receptor gene *ADRB3* in T1D-MSCs. In the bone marrow, nerve fibers that are physically associated with MSCs contribute to the circadian oscillation of HSC mobilization [6, 106, 107]. This event is mainly controlled by sympathetic nervous system (SNS) signaling through the expression of β_3_-adrenergic receptors in bone marrow MSCs, and negatively regulates important HSC maintenance factors, such as CXCL12 and VCAM-1 [6]. T1D patients present bone marrow abnormalities, and are poor HSC mobilizers [108–111]. Indeed, STZ-induced diabetes mice present aberrant SNS signaling in the bone marrow, with impaired expression of CXCL12 [109]. In line with these literature reports, our differential microarray analysis showed downregulation of both HSC maintenance molecules, *CXCL12* and *VCAM1*, in T1D-MSCs.

## Conclusions

Collectively, our findings demonstrate that bone marrow MSCs isolated from newly-diagnosed T1D patients have similar morphology, immunophenotypic profiling, and multipotential differentiation when compared with MSCs from healthy subjects. However, microarray analysis revealed several intrinsic abnormalities in their mRNA expression. MSCs from T1D patients presented enriched canonical pathways related to sympathetic innervation and consequent SNS signaling hyperactivity, with increase of β_3_-adrenergic receptor gene expression. To our knowledge, this is the first report of transcriptional profiling of MSCs isolated from the bone marrow of T1D patients. The implications of these intrinsic genetic alterations in T1D development as well as in the therapeutic efficacy of autologous MSC transplantation require further investigation.

## Abbreviations

α-MEM, minimum essential medium alpha; BRITE, binary relationships of biological entities; C-MSC, bone marrow-derived MSC isolated from healthy donors (controls); CKLF, chemokine-like factor; COX2, cyclooxygenase-2; CXCL12, (C-X-C motif) ligand 12 chemokine; DAVID, Database for Annotation, Visualization and Integrated Discovery; EAE, experimental autoimmune encephalomyelitis; ECM, extracellular matrix; EDTA, ethylenediamine tetraacetic acid; EGFR, epidermal growth factor receptor; EV, expression value; FBS, fetal bovine serum; FC, fold change; FDR, false discovery rate; FGF, fibroblast growth factor; FGFR, fibroblast growth factor receptor; GAD, glutamate decarboxylase; GAPDH, glyceraldehyde 3-phosphate dehydrogenase; GSEA, gene set enrichment analysis; GvHD, graft-versus-host disease; HGF, hepatocyte growth factor, HLA, human leukocyte antigen; HSC, hematopoietic stem cell; IDO, indoleamine 2,3-dioxygenase; IFN-γ, interferon gamma; IFN-γR1, interferon gamma receptor 1; IL, interleukin; IPA, ingenuity pathway analysis; KEGG, Kyoto Encyclopedia of Genes and Genomes; LIMMA, linear models for microarray data; MCP-1, monocyte chemoattractant protein-1; MHC, major histocompatibility complex; MMP, matrix metallopeptidase; MS, multiple sclerosis; MSC, multipotent mesenchymal stromal cell; NOD/SCID, nonobese/severe combined immunodeficiency; PDGF, platelet-derived growth factor; PDGFR, platelet-derived growth factor receptor; RA, rheumatoid arthritis; SLE, systemic lupus erythematosus; SNS, sympathetic nervous system; SSc, systemic sclerosis; STZ, streptozotocin; T1D-MSC, bone marrow-derived MSC isolated from T1D patients; T1D, type 1 diabetes; TGFβ-1, transforming growth factor beta 1; TNF, tumor necrosis factor; TNFR1, tumor necrosis factor receptor superfamily member 1A; Treg, T regulatory cell; VCAM-1, vascular cell adhesion protein 1; VEGF, vascular endothelial growth factor; VLA-4, very late antigen-4

## Additional files

Additional file 1: Table S1. Presenting clinical characteristics of T1D patients at inclusion. (DOCX 49 kb) Additional file 2: Table S2. Showing hierarchical cluster analysis with AU/BP values. The cluster analysis was performed using the pvclust R package. Clusters with AU > 95 % are highlighted by rectangles, which are strongly supported by data. *AU* approximately unbiased *p* value, *BP* bootstrap probability value, *D-PRE* T1D-MSCs, *CTRL* C-MSCs. (TIFF 83966 kb) Additional file 3: Figure S1. Presenting comparison of surface antigen expression in T1D-MSCs and C-MSCs. (DOCX 65 kb) Additional file 4: Figure S2. Showing MSCs present high absolute gene expression of other adhesion-related molecules. Genes with multiple probes are represented by the median value, in T1D-MSCs (*Diabetic*) and C-MSCs (*Control*). Only genes with high absolute gene expression are shown. The EV was log_2_-transformed. (TIFF 108909 kb) Additional file 5: Figure S3. Showing T1D-MSCs have higher motility after in-vitro migration assay. (A) Representative images of transwell migration assay using 50 % FBS as chemoattractant, showing Giemsa-stained MSCs from healthy individuals (*C-MSCs*, *left*) and from T1D patients (*T1D-MSCs*, *right*). (B) The fraction of cells that migrated across 8 μm diameter pores over 6 h was counted in seven different fields using light microscopy. Bars represent means ± SEM. **p* < 0.05. 200× magnification. (TIFF 50715 kb) Additional file 6: Table S3. Presenting DAVID analysis of the upregulated genes. (DOCX 53 kb)
