# Supplementary material for: Transcriptional profiling reveals intrinsic mRNA alterations in multipotent mesenchymal stromal cells isolated from bone marrow of newly-diagnosed type 1 diabetes patients
Source: Stem Cell Res Ther. 2016 Jul 12;7:92. doi: 10.1186/s13287-016-0351-y (PMC4942931; doi:10.1186/s13287-016-0351-y)
Supplement: Additional file 1: Table S1. — Presenting clinical characteristics of T1D patients at inclusion. (DOCX 49 kb) [file 13287_2016_351_MOESM1_ESM.docx]

**Supplementary Table 1**

**Type 1 diabetes patient’s clinical characteristics at inclusion**

| **Patient ID** | **anti-GAD65 (U/mL)** | **Glycemia (mg/dL)** |
| --- | --- | --- |
| 1 | 1.1 | 381 |
| 2 | 22 | 321 |
| 3 | 51 | 404 |
| 4 | 17 | 504 |
| 5 | 4 | 391 |
| 6 | 48 | 314 |
| 7 | 102 | 330 |
| 8 | 44 | 612 |
| 9 | 11 | 130 |
| 10 | 11 | 581 |
| 11 | 24 | 269 |
| 12 | 37 | 273 |
| 13 | 21.1 | 291 |
| 14 | 5.3 | 384 |
| 15 | 2.9 | 236 |
| 16 | 7 | 324 |
| 17 | 16 | 793 |
| 18 | 1.1 | 439 |
| 19 | 29 | 485 |
| 20 | 10 | 390 |
| 21 | 19.5 | 250 |

Serum levels of anti-GAD antibodies were measured by radioimmunoassay using commercial kits (RSR Limited, Cardiff, UK) and the results were considered positive if greater than 1 U/mL.
