# Supplementary material for: Transcriptional profiling reveals intrinsic mRNA alterations in multipotent mesenchymal stromal cells isolated from bone marrow of newly-diagnosed type 1 diabetes patients
Source: Stem Cell Res Ther. 2016 Jul 12;7:92. doi: 10.1186/s13287-016-0351-y (PMC4942931; doi:10.1186/s13287-016-0351-y)
Supplement: Additional file 2: Table S2. — Showing hierarchical cluster analysis with AU/BP values. The cluster analysis was performed using the pvclust R package. Clusters with AU > 95 % are highlighted by rectangles, which are strongly supported by data. AU approximately unbiased p value, BP bootstrap probability value, D-PRE T1D-MSCs, CTRL C-MSCs. (TIFF 83966 kb) [file 13287_2016_351_MOESM2_ESM.docx]

**Supplementary Table 2**

**Comparison of surface antigen expression in MSCs derived from type 1 diabetes patients (T1D-MSCs) and from healthy individuals (C-MSCs)**

| **Marker** | **T1D-MSCs (n=11)** | **C-MSCs (n=10)** |
| --- | --- | --- |
| CD90 | 98.3 ± 1.16 | 97.87 ± 1.54 |
| CD73 | 85.57 ± 10.98 | 84.18 ± 13.52 |
| CD105 | 76.04 ± 15.99 | 86.78 ± 6.09 |
| CD44 | 65.11 ± 25.16 | 80.30 ± 8.85 |
| CD29 | 94.82 ± 2.43 | 93.10 ± 5.28 |
| CD49e | 91.29 ± 5.98 | 86.08 ± 11.86 |
| CD13 | 96.64 ± 3.15 | 95.58 ± 3.78 |
| HLA-ABC | 66.34 ± 24.44 | 74.15 ± 15.12 |
| CD54 | 29.38 ± 13.05 | 33.96 ± 24.22 |
| STRO-1 | 9.66 ± 8.47 | 10.02 ± 12.91 |
| CD45 | 0.08 ± 0.15 | 0.27 ± 0.35 |
| CD34 | 0.32 ± 0.69 | 0.36 ± 0.43 |
| CD14 | 0.06 ± 0.10 | 0.62 ± 1.03 |
| HLA-DR | 0.33 ± 0.36 | 0.36± 0.43 |
| CD51/61 | 2.52 ± 3.19 | 2.57 ± 2.49 |
| KDR | 0.74 ± 0.66 | 1.10 ± 0.89 |
| CD106 | 11.48 ± 15.27 | 23.02 ± 23.01 |

The table shows mean values of the percentage of positive cells ± standard deviation to the total number of cells analyzed, as determined by flow cytometry. Non-significant differences were observed between groups (P > 0.05 by Manny-Whitney test).
