# Supplementary figures and images for: Transcriptional profiling reveals intrinsic mRNA alterations in multipotent mesenchymal stromal cells isolated from bone marrow of newly-diagnosed type 1 diabetes patients
Source: Stem Cell Res Ther. 2016 Jul 12;7:92. doi: 10.1186/s13287-016-0351-y (PMC4942931; doi:10.1186/s13287-016-0351-y)

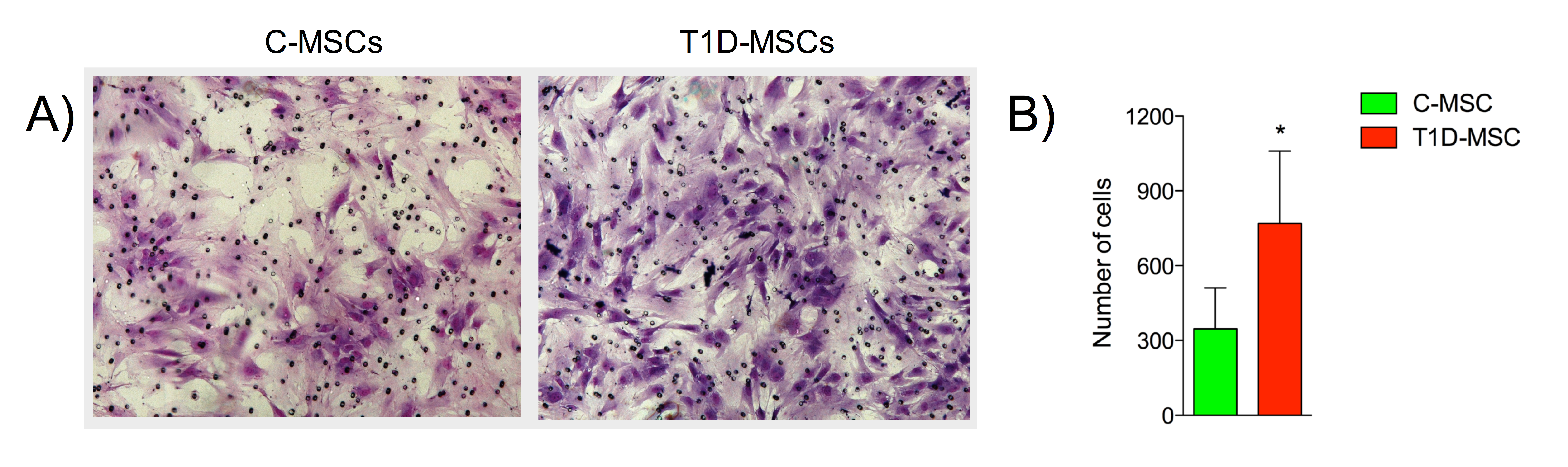

Supplement: Additional file 5: Figure S3. — Showing T1D-MSCs have higher motility after in-vitro migration assay. (A) Representative images of transwell migration assay using 50 % FBS as chemoattractant, showing Giemsa-stained MSCs from healthy individuals (C-MSCs, left) and from T1D patients (T1D-MSCs, right). (B) The fraction of cells that migrated across 8 μm diameter pores over 6 h was counted in seven different fields using light microscopy. Bars represent means ± SEM. *p < 0.05. 200× magnification. (TIFF 50715 kb) [file 13287_2016_351_MOESM5_ESM.tiff]
