# Supplementary material for: Transcriptional profiling reveals intrinsic mRNA alterations in multipotent mesenchymal stromal cells isolated from bone marrow of newly-diagnosed type 1 diabetes patients
Source: Stem Cell Res Ther. 2016 Jul 12;7:92. doi: 10.1186/s13287-016-0351-y (PMC4942931; doi:10.1186/s13287-016-0351-y)
Supplement: Additional file 6: Table S3. — Presenting DAVID analysis of the upregulated genes. (DOCX 53 kb) [file 13287_2016_351_MOESM6_ESM.docx]

**Supplementary Table 3**

**DAVID analysis of the upregulated genes**

| **Term** | **Fold Enrichment** | **Benjamini** |
| --- | --- | --- |
| Neuroactive ligand-receptor interaction | 1.642540565 | 0.178684527 |
| Tight junction | 1.751435132 | 0.526229546 |
| Antigen processing and presentation | 2.0028962 | 0.407861918 |
| Calcium signaling pathway | 1.555725524 | 0.582810101 |
| MAPK signaling pathway | 1.428370787 | 0.515740127 |
| Cyanoamino acid metabolism | 5.587912088 | 0.560608309 |
| Maturity onset diabetes of the young | 2.738076923 | 0.61119605 |
| Selenoamino acid metabolism | 2.632766272 | 0.62801129 |
| Long-term depression | 1.842391304 | 0.597802632 |
| Type II diabetes mellitus | 2.080605565 | 0.564595373 |

Only shown the first ten KEGG Pathway.
